# A cellulose synthase-derived enzyme catalyses 3-*O*-glucuronosylation in saponin biosynthesis

**DOI:** 10.1038/s41467-020-19399-0

**Published:** 2020-11-16

**Authors:** Soo Yeon Chung, Hikaru Seki, Yukiko Fujisawa, Yoshikazu Shimoda, Susumu Hiraga, Yuhta Nomura, Kazuki Saito, Masao Ishimoto, Toshiya Muranaka

**Affiliations:** 1 Department of Biotechnology, Graduate School of Engineering, Osaka University, 2-1, Yamadaoka, Suita, Osaka 565-0871 Japan; 2 RIKEN Center for Sustainable Resource Science, 1-7-22 Suehiro-cho, Tsurumi-ku, Yokohama, Kanagawa 230-0045 Japan; 3 Institute of Crop Science, NARO, 2-1-2 Kannondai, Tsukuba, Ibaraki 305-8518 Japan; 4 Institute of Agrobiological Sciences, NARO, 1-2 Owashi, Tsukuba, Ibaraki 305-8634 Japan; 5 Graduate School of Pharmaceutical Sciences, Chiba University, 1-8-1 Inohana, Chuo-ku, Chiba 260-8675 Japan; 6 Present Address: RIKEN Center for Sustainable Resource Science, 2-1 Hirosawa, Wako, Saitama 351-0198 Japan

**Keywords:** Transferases, Metabolic engineering, Molecular engineering in plants, Secondary metabolism

## Abstract

Triterpenoid saponins are specialised metabolites distributed widely in the plant kingdom that consist of one or more sugar moieties attached to triterpenoid aglycones. Despite the widely accepted view that glycosylation is catalysed by UDP-dependent glycosyltransferase (UGT), the UGT which catalyses the transfer of the conserved glucuronic acid moiety at the C-3 position of glycyrrhizin and various soyasaponins has not been determined. Here, we report that a cellulose synthase superfamily-derived glycosyltransferase (CSyGT) catalyses 3-*O-*glucuronosylation of triterpenoid aglycones. Gene co-expression analyses of three legume species (*Glycyrrhiza uralensis, Glycine max*, and *Lotus japonicus*) reveal the involvement of CSyGTs in saponin biosynthesis, and we characterise CSyGTs in vivo using *Saccharomyces cerevisiae*. *CSyGT* mutants of *L. japonicus* do not accumulate soyasaponin, but the ectopic expression of endoplasmic reticulum membrane–localised CSyGTs in a *L. japonicus* mutant background successfully complement soyasaponin biosynthesis. Finally, we produced glycyrrhizin de novo in yeast, paving the way for sustainable production of high-value saponins.

## Abstract (web summary)

Saponins such as glycyrrhizin, a natural sweetener found in licorice root, are a class of triterpenoids synthesized that are characterized by a glucoronic acid moiety at the C-3 position. Here the authors show that saponin glucuronosylation is catalyzed by cellulose-synthase like enzymes and reconstitute glycyrrhizin synthesisin yeast.

## Introduction

Triterpenoid saponins constitute a vast class of natural products that are considered to be high-value compounds due to their immense structural diversity and a wide range of biological activities^1^. *Glycyrrhiza uralensis* (liquorice) is one of the most economically important medicinal plants^2^, and its major active compound, glycyrrhizin, has many pharmacological properties, such as anti-inflammatory^3^, anti-ulcer^4^ and hepatoprotective^5^ activities. Glycyrrhizin is used worldwide as a natural sweetener and food additive because it is 150 times sweeter than sucrose^6^. *G. uralensis* produces other structurally different triterpenoids, including soyasaponins^7^, betulinic acid and oleanolic acid^8^. Whereas glycyrrhizin is found only in *Glycyrrhiza* species, soyasaponins are prevalent in legumes, and are particularly abundant in soybeans (*Glycine max*). Soyasaponins also have several beneficial effects on human health due to their anti-carcinogenic, anti-oxidant and cardioprotective activities^9^. However, some soyasaponins are viewed as undesirable because of their bitter, astringent aftertaste^10^. Hence, the biosynthetic pathway of triterpenoid saponins has been studied extensively to inform the establishment of a heterologous system for the production of commercially valuable triterpenoid saponins and the engineering of a pathway for crop-quality control.

**Fig. 1:**
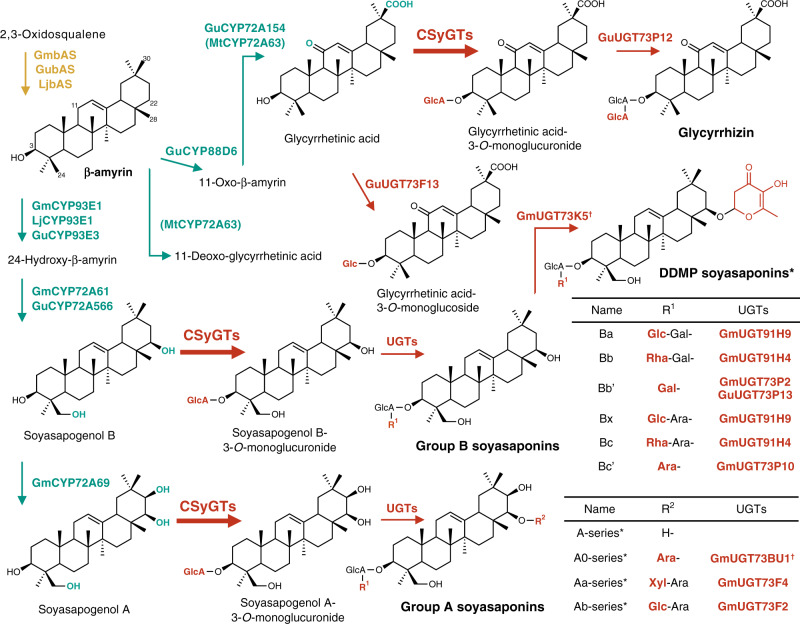
Proposed biosynthetic pathways of oleanane-type triterpenoid saponins catalysed by characterised enzymes in *Glycine max*, *Glycyrrhiza uralensis* and *Lotus japonicus*. The yellow arrow indicates the cyclisation reaction catalysed by β-amyrin synthase (bAS), green arrows show oxidation reactions catalysed by cytochrome P450 monooxygenases (P450s) and red arrows indicate glycosylation reactions catalysed by UDP-dependent glycosyltransferases (UGTs). The reaction sites are indicated by the corresponding colours. MtCYP72A63 is in parentheses because its catalytic reaction is represented by the results of enzyme assays conducted with exogenous substrates. Bold red arrows indicate glycosylation reactions catalysed by the novel cellulose-synthase-derived glycosyltransferase (CSyGT) characterised in this paper. Gm *G*. *max*; Gu *G*. *uralensis*, Lj *L*. *japonicus*, Mt *Medicago truncatula*, Ara arabinose, Gal galactose, Glc glucose, GlcA glucuronic acid, Rha rhamnose, Xyl xylose. *The glycosylation pattern of R^1^ is identical to that of group B soyasaponins. ^†^Unpublished data.

Triterpenoid saponin biosynthesis begins with cyclisation of the common precursor 2,3-oxidosqualene by oxidosqualene cyclases (OSCs)^11^ into various triterpene scaffolds. These triterpene scaffolds undergo site-specific oxidation catalysed by cytochrome P450 monooxygenase (P450s), forming diverse triterpenoid aglycones or non-glycosylated forms. Lastly, glycosylation reactions catalysed by the glycosyltransferase 1 superfamily, often referred as UDP-dependent glycosyltransferases (UGTs), yield structurally diverse triterpenoid saponins. Glycyrrhizin and soyasaponins are oleanane-type triterpenoid saponins, which are derived from β-amyrin, and most of their biosynthetic enzymes have been identified (Fig. 1)^12–17^. However, the enzyme that catalyses the transfer of the conserved glucuronosyl moiety at the C-3 position of the aglycones has not yet been determined. The sugar chain of triterpenoid saponins is thought to affect these products’ activity^18^. For instance, the glucuronic acid moiety in glycyrrhizin is crucial for its sweet taste, and glycyrrhetinic acid monoglucuronide is reported to be a more potent sweetener than glycyrrhizin^19^. All four soyasaponin groups (A, B, E and DDMP) have the same oligosaccharide composition at the C-3 position, despite their structural differences^20^. For example, soyasaponins in groups A, B and E are glycosides of the corresponding aglycones (soyasapogenol A, B and E), and soyasaponins in group DDMP differ from those in group B only by a DDMP (2,3-dihydro-2,5-dihydroxy-6-methyl-4H-pyran-4-one) moiety attached to the C-22 position. Although the exact biological roles of these soyasaponins and the effects of oligosaccharides on these roles are not understood, the conservation of C-3 sugar chain composition among different groups of soyasaponins and the ubiquitous distribution of these compounds in planta imply the biological importance of this chain.

We have characterised numerous UGTs as potential candidates for the target glucuronosyltransferase. However, the massive number of UGTs found per plant genome and the low degree of correlation between substrate selectivity and the primary UGT sequence have made identification of the target UGT difficult^21^. Although GuUGAT^22^ (UGT73B27) reportedly catalyses continuous two-step glucuronosylation to yield glycyrrhizin directly from its aglycone, glycyrrhetinic acid, our recent study on UGT73P12^14^, which catalyses only the second glucuronosylation, strongly suggests that a separate enzyme is responsible for the first glucuronosylation. Here, we demonstrate that a cellulose-synthase superfamily-derived glycosyltransferase (CSyGT) catalyses the transfer of glucuronic acid from UDP-glucuronic acid to the C-3 position of oleanane-type triterpenoid aglycones. Filling in the last piece of the glycyrrhizin biosynthetic pathway, we successfully produced glycyrrhizin de novo in yeast from a simple sugar. Our study challenges the conventional theory that specialised plant metabolites are glycosylated by UGTs and provides a foundation for the microbial production of glycyrrhizin, in a stable, cost-efficient manner.

## Results

### CSyGTs catalyse triterpenoid 3-*O*-glucuronosylation

**Fig. 2:**
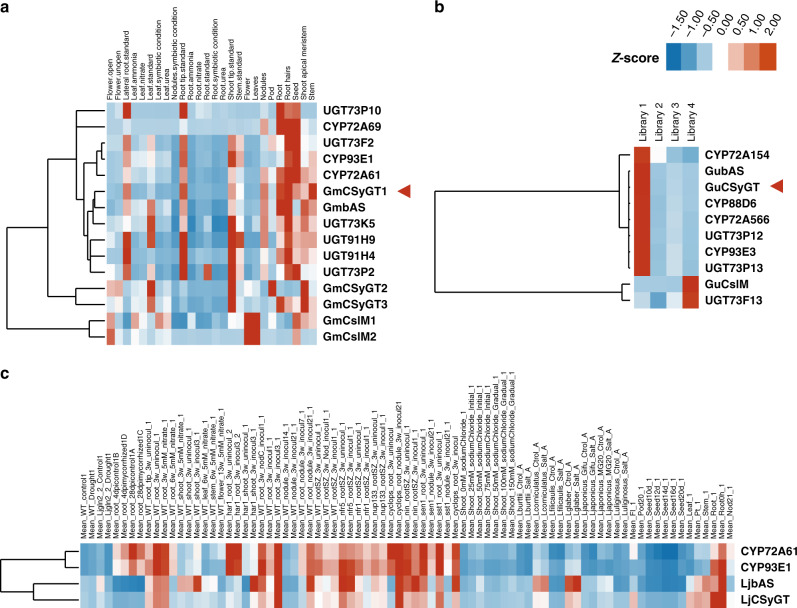
Gene co-expression analyses of CSyGTs in *G*. *max*, *G*. *uralensis* and *L*. *japonicas*. All heatmaps are in hierarchical clustering of expression profiles and expression levels are normalised across conditions. **a** *G. max* expression profiles retrieved from Phytozome. **b** UniGene expression profiles of *G*. *uralensis*. Library 1 extracted from roots of 308–19 (high glycyrrhizin-producing) strain in June; Library 2 from roots of 308–19 strain in December; Library 3 from roots of 87–458 (low glycyrrhizin-producing) strain in June; Library 4 from leaves of 308–19 strain in June. **c** Gene expression profiles of *L*. *japonicus* retrieved from Lotus Base.

To identify the enzyme that catalyses the first glucuronosylation, we performed gene co-expression analyses using a database of co-functional networks for soybean^23^. We observed that *Glyma.06G324300* (*GmCSyGT1*), a member of the cellulose-synthase superfamily, had an expression pattern strongly correlated with that of genes involved in soyasaponin biosynthesis (Fig. 2a). We then used GmCSyGT1 as a query to mine for homologues in the *G. uralensis*^24^ and *L. japonicus*^25^ transcriptomes using the Basic Local Alignment Search Tool (blast). GuCSyGT (Glyur003152s00037491) and LjCSyGT (Lj3g3v1981230) had an amino acid sequence identity of >80%, and their expression patterns were highly correlated with those of genes involved in saponin biosynthesis (Fig. 2b, c).

**Fig. 3:**
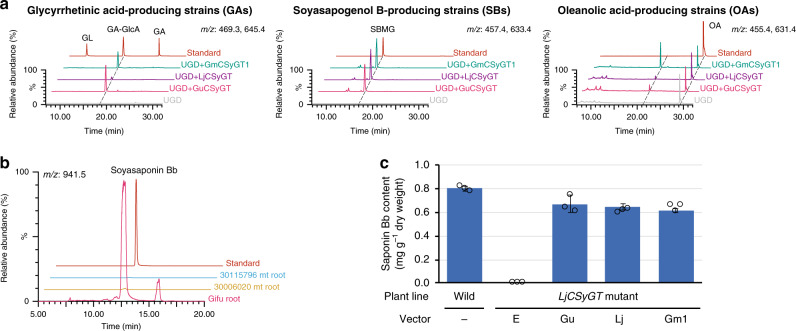
Functional characterisation of CSyGTs in yeast and in planta. **a** Overlays of LC–MS chromatograms obtained by selected-ion monitoring (SIM) of the theoretical *m/z* values of the compounds of interest. Chromatograms of monoglucuronides produced in vivo in transformed glycyrrhetinic acid-producing strains (GA), soyasapogenol B-producing strains (SB) and oleanolic acid-producing strains (OA) with GuCSyGT, LjCSyGT and GmCSyGT1. **b** Soyasaponin Bb accumulation in the roots of wild-type (Gifu) and *LjCSyGT* mutant lines (Supplementary Fig. 2). **c** Soyasaponin Bb accumulation in the roots of wild-type and *LjCSyGT* mutants transformed with GuCSyGT (Gu), LjCSyGT (Lj), GmCSyGT1 (Gm1) or empty vector (E). Three biologically independent replicates were performed. Error bars represent SDs (*n* = 3).

All three CSyGTs were predicted to be transmembrane proteins with multiple transmembrane helices: two at the N terminus and three or more at the C terminus (Supplementary Fig. 1)^26^. For functional analyses, we introduced each CSyGT into previously engineered triterpenoid aglycone-producing yeast strains (GA, glycyrrhetinic acid; SB, soyasapogenol B^27^; OA, oleanolic acid^28^) with *UDP-glucose dehydrogenase* (*UGD*)^29^, to enable synthesis of UDP-glucuronic acid from endogenous UDP-glucose. The resulting strains (GA0–3, SB0–3 and OA0–3) were cultured and their metabolites were analysed by liquid chromatography–mass spectrometry (LC–MS). All of the CSyGT-expressing strains produced the corresponding monoglucuronides (Fig. 3a). While all three had similar levels of catalytic activity for soyasapogenol B, there were some differences for glycyrrhetinic and oleanolic acids, implying different substrate selectivity among the three CSyGTs. The low catalytic activity of LjCSyGT for glycyrrhetinic and oleanolic acids is consistent with the triterpenoid profile of *L. japonicus*, which does not accumulate glycyrrhizin and accumulates oleanolic acid as a non-glycosylated form^30^.

Next, we analysed mutants of *L. japonicus* harbouring *Lotus retrotransposon 1* (*LORE1*)^31^ in *LjCSyGT* to confirm CSyGT function in planta. The *L. japonicus LjCSyGT* mutants were deficient in soyasaponin Bb by LC–MS (Fig. 3b). Then, we generated hairy roots of *L. japonicus* mutants transformed with *GuCSyGT*, *LjCSyGT* and *GmCSyGT1* via *Agrobacterium rhizogenes*-mediated transformation, and quantified their soyasaponin Bb content by LC–MS. Introduction of *CSyGTs* successfully complemented soyasaponin Bb biosynthesis, confirming that CSyGTs are functional in planta (Fig. 3c).

### CSyGTs localise to the endoplasmic reticulum

**Fig. 4:**
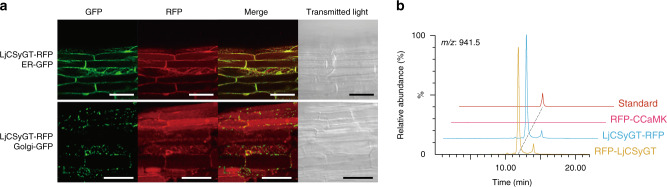
Subcellular localisation of LjCSyGT. **a** Confocal image of LjCSyGT-RFP co-expressed in hairy roots of the *LjCSyGT* mutant lines with an endoplasmic reticulum (ER) marker and a Golgi marker. Scale bar, 50 µm. LjCSyGT-RFP shows the characteristic web-like pattern of the ER network, suggesting localisation to the ER. **b** Soyasaponin Bb accumulation in transgenic roots of *LjCSyGT* mutant lines expressing RFP-LjCSyGT, LjCSyGT-RFP or RFP-CCaMK^49^ (calcium/calmodulin-dependent protein kinase is known to localise to the nucleus) as a control.

As CSyGTs are predicted to be transmembrane enzymes, whereas canonical UGTs are cytosolic, we decided to analyse the subcellular localisation of CSyGTs. We obtained transgenic hairy roots of *L. japonicus LjCSyGT* mutants expressing LjCSyGT fused to red fluorescent protein (RFP) together with each organelle marker [endoplasmic reticulum (ER) or Golgi markers fused to green fluorescent protein (GFP)]. We used a mutant line to avoid conflict with endogenous LjCSyGT and to confirm that RFP-fused CSyGTs remained functional. We observed that LjCSyGT-RFP had an ER network with a characteristic web-like pattern, as shown by the ER-GFP marker (Fig. 4a). In comparison, the characteristic spotty features depicted with Golgi-GFP did not completely match the fluorescent pattern of LjCSyGT-RFP. We then analysed the saponin content of the obtained transgenic hairy roots, and confirmed that LjCSyGT with N- or C-terminal RFP fusion retained its glucuronosyltransferase activity in planta (Fig. 4b and Supplementary Fig. 3). With these results, we demonstrated that CSyGTs are functional glucuronosyltransferases localised to the ER.

### CSyGTs have diverged functionally from CslM

**Fig. 5:**
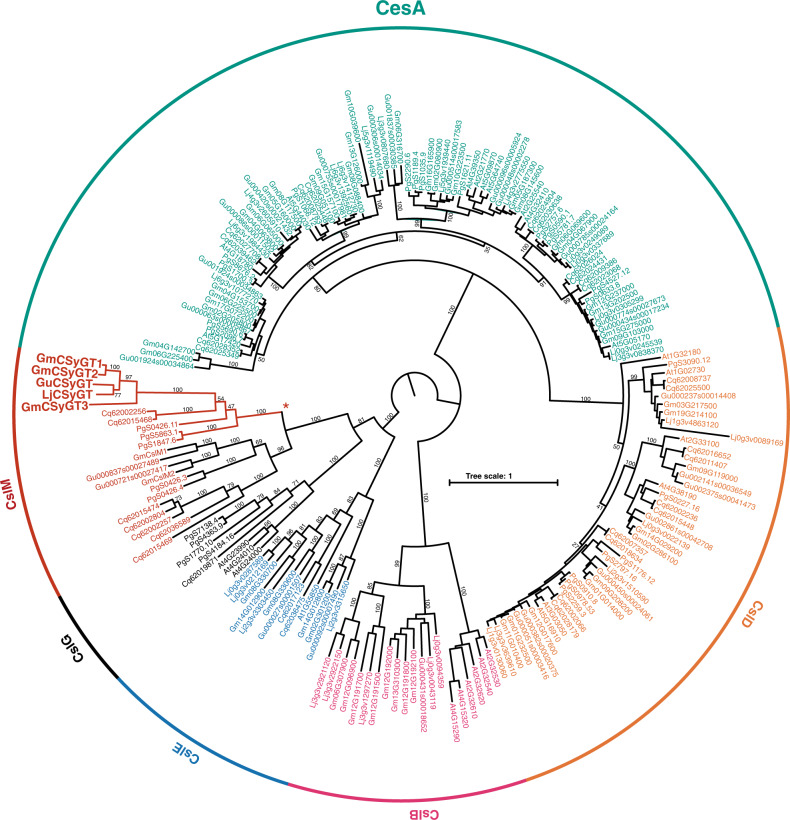
Phylogenetic analyses of the cellulose-synthase superfamily in sampled dicots. Best-scoring maximum likelihood (ML) tree constructed using RAxML. Numbers are the bootstrap values (%) from 1,000 replicates. *CSyGTs are clustered together, expanding from other CslMs. At *Arabidopsis thaliana,* Cq *Chenopodium quinoa,* Gm *Glycine max*, Gu *Glycyrrhiza uralensis,* Lj *Lotus japonicus*, Pg *Panax ginseng*.

To determine whether glucuronosyltransferase activity is unique to CSyGTs among the cellulose-synthase superfamily, we performed phylogenetic analyses based on the transcriptomes of *Arabidopsis thaliana*, *Chenopodium quinoa*, *G. max*, *G. uralensis*, *L. japonicus*, and *Panax ginseng* (Fig. 5). *C. quinoa* and *P. ginseng* were included as both produce triterpenoid saponins with the conserved glucuronic acid moiety at the C-3 position and are phylogenetically distant from the Leguminosae^32, 33^. The cellulose-synthase superfamily is the glycosyltransferase 2 superfamily, and consists of the cellulose-synthase family (CesA), which catalyses cellulose biosynthesis, and ten cellulose-synthase-like families (*Csl A*–*H*, *J* and *M*) that are predicted to be involved in the biosynthesis of hemicellulose^34^. Unlike UGTs, which have a GT-B fold in their crystal structure, the cellulose-synthase superfamily has a GT-A fold^35^. All CSyGTs were classified into the cellulose-synthase-like M subfamily (CslM), a newly discovered eudicot expansion of CslJ, a monocot clade capable of synthesising (1,3;1,4)-β-glucan in the cell wall^34^. According to the same study, CslMs were incapable of synthesising (1,3;1,4)-β-glucans and there has been no further functional characterisation of CslMs. Next, we repeated the analyses with the inclusion of CslMs from other angiosperms (Supplementary Fig. 4)^34^. CslMs were widely distributed among eudicots, and multiple genes were found in most plant species. The CslM subfamily was divided into two distinct clades, one including CSyGTs and the other not including CSyGTs. Interestingly, species with multiple CslMs had genes in each clade, irrespective of their phylogenetic relationship. For instance, *G. max* had two homologues in the CSyGT clade (GmCSyGT2 and GmCSyGT3) and two homologues in the non-CSyGT clade (GmCslM1 and GmCslM2).

**Fig. 6:**
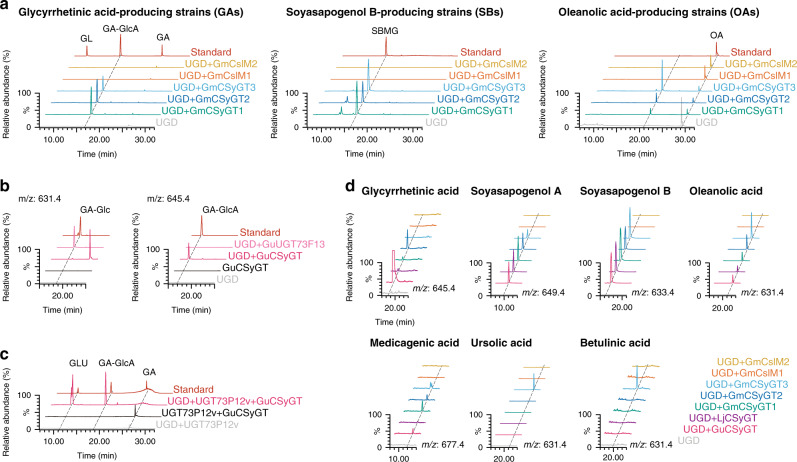
In vivo assays of CSyGT and GmCslM activity using a yeast-expression system. All overlays of chromatograms were analysed by LC–MS with selected-ion monitoring (SIM) of the theoretical *m/z* values of the compounds of interest. Signals were compared to authentic standards (standard) if available. **a** Chromatograms of in vivo-produced monoglucuronides by transformed triterpenoid aglycone-producing strains with GmCSyGT1–3, GmCslM1 or GmCslM2. **b** Chromatograms of products of transformed glycyrrhetinic acid-producing strains (GAs), selected based on the theoretical *m/z* values of 631.4 (left) and 645.4 (right) of glycyrrhetinic acid monoglucoside and monoglucuronide, respectively. **c** Chromatogram of in vivo-produced glucoglycyrrhizin (GLU, GA–GlcA–Glc) and intermediates by transformed glucoglycyrrhizin-producing platform strains (GLU). **d** Results of in vivo substrate-feeding assays of CSyGTs and GmCslMs. Structures of the substrates and full-length LC–MS chromatograms are shown in Supplementary Fig. 6. GA glycyrrhetinic acid (*m/z* 469.3), GA–GlcA glycyrrhetinic acid-3*-O-*monoglucuronide (*m/z* 645.4), GA–Glc glycyrrhetinic acid-3-*O-*monoglucoside (*m/z* 631.4), GL glycyrrhizin (*m/z* 821.4), GLU glucoglycyrrhizin (*m/z* 807.4), SBMG soyasapogenol B-3-*O-*monoglucuronide (*m/z* 633.4), OA oleanolic acid (*m/z* 631.4).

To obtain insight into the functional differentiation of CSyGTs from CslMs, we subjected GmCSyGTs and GmCslMs to the same enzyme assays. We introduced each homologue into triterpenoid aglycone-producing yeast strains (GA, SB and OA), yielding the GA4–7, SB4–7 and OA4–7 strains. Two of the homologues, GmCSyGT2 and 3, showed glucuronosyltransferase activity, while the other two, GmCslM1 and 2, did not (Fig. 6a). These results imply that CSyGTs have diverged functionally from CslMs by acquiring glucuronosyltransferase activity against triterpenoid aglycones, and that CslMs may retain catalytic activity related to cell wall biosynthesis. Moreover, the proportion of amino acid sequence identity between GmCSyGT1 and GmCslM1 was only 43%, whereas that between GmCSyGT1 and GmCSyGT3 was 72%.

### CSyGTs are specific to UDP-glucuronic acid

In the course of this research, we also identified GuUGT73F13, which catalyses 3-*O*-glucosylation of glycyrrhetinic acid. We expressed GuUGT73F13 with UGD in a glycyrrhetinic acid-producing strain (GA8) and compared its metabolite against that of a GuCSyGT-expressing strain. Moreover, we produced a GuCSyGT-expressing glycyrrhetinic acid-producing strain without UGD expression (GA9) to assess its catalytic activity against UDP-glucose. According to the extracted ion chromatogram with an *m/z* value of 631.4 (glycyrrhetinic acid monoglucoside), a peak corresponding to glycyrrhetinic acid-3-*O-*monoglucoside was detected in the GuUGT73F13, but not the GuCSyGT-expressing strain (Fig. 6b). A peak at a later retention time was detected only in the presence of UGD, which was later identified as 11-deoxo-glycyrrhetinic acid (intermediate) monoglucuronide (Supplementary Fig. 5). This result shows that sufficient UDP-glucose is present for glycosylation by GuUGT73F13 in the presence of UGD, and thus that the absence of glycyrrhetinic acid monoglucoside in the GuCSyGT-expressing strain is not due to the depletion of UDP-glucose by UGD. Next, we engineered a glucoglycyrrhizin-producing platform yeast strain (GLU) expressing β-amyrin synthase, CYP88D6, CYP72A63 and UGT73P12v. UGT73P12 catalyses the second glucuronosylation in glycyrrhizin biosynthesis, and its natural variant UGT73P12v, obtained from glucoglycyrrhizin-producing *G. uralensis* 83–555 strain, has lost its specificity for UDP-glucuronic acid^14^. Instead, UGT73P12v catalyses transfer of glucose, yielding glucoglycyrrhizin. GuCSyGT with and without UGD was introduced into GLU, resulting in GLU0–2. LC–MS analyses showed that co-expression of GuCSyGT and UGT73P12v in the presence of UGD yielded glucoglycyrrhizin (Fig. 6c). These results demonstrate that GuCSyGT preferentially selects UDP-glucuronic acid as the sugar donor even in the presence of UDP-glucose.

### Substrate scope of CSyGTs

To investigate the substrate range of CSyGTs, we introduced all CSyGTs and GmCslMs into wild-type yeast (INV*Sc*I) with UGD and cultured the resulting strains (FA0–7) in medium supplemented with 10 µM of various substrates (Supplementary Fig. 6). The substrates included various oleanane-type triterpenoid saponins, other triterpenoid saponin types with different backbones (ursane and lupane types) and some flavonoids (another major class of specialised plant metabolite). All CSyGTs showed catalytic activity against oleanane-type triterpenoid aglycones, consistent with the results of in vivo enzyme assays (Fig. 6d). The peak intensity of putative medicagenic acid monoglucuronide was lower than those of other substrate monoglucuronides, possibly due to the presence of a carboxyl group near the catalytic site, 3-OH. In addition, GmCSyGT3 showed catalytic activity against the triterpenoid aglycones, ursolic acid and betulinic acid, which are derived from α-amyrin and lupeol triterpene scaffolds, respectively. However, GmCSyGT3 did not show glucuronosyltransferase activity against β-boswellic acid, another α-amyrin-derived ursane-type triterpenoid aglycone. As β-boswellic acid also has a carboxyl group near 3-OH, the result is consistent with that of medicagenic acid. CSyGTs did not show significant catalytic activity against kaempferol and genistein. Although putative peaks (indicated by dashed lines) are found among some CSyGTs for β-amyrin and liquiritigenin, these compounds are unlikely to be the major catalytic substrates (Supplementary Fig. 6). In comparison, GmCslMs did not show glucuronosyltransferase activity against any substrate.

### De novo production of glycyrrhizin

**Fig. 7:**
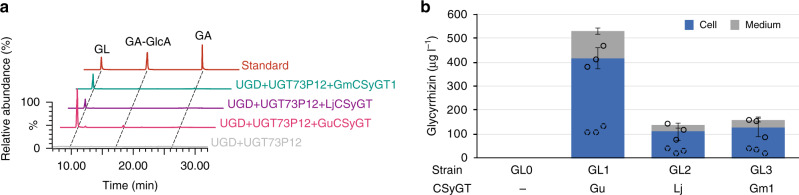
De novo production of glycyrrhizin in yeast. **a** Overlay of LC–MS chromatogram showing in vivo production of glycyrrhizin in the yeast strain GL0–3. Signals were compared to authentic standards. Chromatograms were selected based on the theoretical *m/z* values of glycyrrhetinic acid (GA, 469.3), glycyrrhetinic acid-3*-O-*monoglucuronide (GA–GlcA, 645.4) and glycyrrhizin (GL, GA–GlcA–GlcA, 821.4). **b** Glycyrrhizin production by GL0–3. The amount of glycyrrhizin was measured separately from collected cells (circles) and medium (dashed circles) 5 days after induction. Error bars represent SDs (*n* = 3).

Finally, we engineered a glycyrrhizin-producing yeast strain by reconstructing the whole glycyrrhizin biosynthetic pathway, including GuCSyGT, LjCSyGT and GmCSyGT1. The glycyrrhizin-producing yeast strains (GL1–3) cultured for 5 days produced 528.1, 136.2 and 158.7 µg l^–1^ glycyrrhizin, respectively (Fig. 7). When cultured for 10 days, glycyrrhizin production increased to 791, 216.2 and 225.3 µg l^–1^, respectively. There was not much difference in the production of glycyrrhizin between LjCSyGT and GmCSyGT1, despite the difference observed in Fig. 3a. This may be due to stronger negative feedback to LjCSyGT by its product, glycyrrhetinic acid-3-*O*-glucuronide. Since the product is quickly converted into glycyrrhizin in GL strains, the effect of feedback may have been reduced, leading to productivity similar to that of GmCSyGT1. In addition, a significant quantity of glycyrrhizin was secreted into the medium (Fig. 7b). Because the negative control (GL0, all enzymes present, except CSyGT) did not accumulate glycyrrhetinic acid, we assume that glycosylation reduced the toxicity of glycyrrhetinic acid, and hence enhanced the accumulation of glycyrrhizin.

## Discussion

In conclusion, we identified a new enzyme, CSyGT, that catalyses the first glucuronosylation of oleanane-type aglycones. Because GmCSyGT3, positioned between CSyGT and non-CSyGT clades, catalyses more diverse substrates than other CSyGTs, we speculate that other CSyGTs have evolved to be more specific for oleanane-type triterpenoid saponins. However, the substrate selectivity of each CSyGT should be quantified with detailed enzyme kinetic analysis to allow for accurate comparison.

We note that Jozwiak et al.^36^ published a paper similar to ours while this work was under revision. They discovered that SOAP5, co-expressed with saponin biosynthetic enzymes in spinach (*Spinacia oleracea*), catalyses the 3-*O-*glucuronosylation of medicagenic acid. They also characterised homologues from diverse plants, including *L. japonicus, G. uralensis, G. max, Medicago sativa, Beta vulgaris* and *C. quinoa*, and observed the 3-*O-*glucuronosylation of medicagenic acid by transient expression with other medicagenic acid biosynthetic enzymes in *Nicotiana benthamiana* leaves. However, they classified SOAP5 and its homologues in the CslG subfamily, whereas we adopted a new phylogeny for the cellulose-synthase gene superfamily. The GmCsl they characterised corresponds to GmCSyGT3, and the homologue found in quinoa corresponds to Cq62015468 in our phylogenetic analyses (Fig. 5). We observed only 33% similarity between the amino acid sequences of Cq62015468, which is located in the CSyGT clade, and Cq62019871, classified as CslG in our analyses. As *A. thaliana* lacks CslM, we recommend classification based on phylogenetic analysis rather than annotation based on *A. thaliana* Csls.

Although the catalytic mechanism of the CSyGTs in triterpenoid glycosylation is unknown, we hypothesise that the transmembrane topology of CSyGTs is crucial. Our study of the *G. uralensis* UGTs, UGT73P12 (which catalyses the second glucuronosylation) and GuUGT73F13 (which catalyses 3-*O-*glucosylation of glycyrrhetinic acid), showed that neither glucuronosyltransferase activity nor glycosyltransferase activity at the C-3 position is a problematic reaction for UGTs to catalyse. Yet, the first glucuronosyltransferase at the C-3 position is catalysed by enzymes in a totally different superfamily. As plant P450s and their redox partners NADPH-cytochrome P450 reductases (CPRs) are anchored to the ER^37^, whereas most plant UGTs are cytosolic^38^, ER-localised transmembrane CSyGTs may also play a structural role as a hub for the formation of a triterpenoid saponin metabolon to facilitate channelling of mono-glucuronosylated triterpenoids to the UGT for further glycosylation. Metabolon formation and metabolic channelling is observed in other specialised plant metabolites, such as flavonoids and cyanogenic glycosides^39^. In addition, CesA members are reported to localise to the plasma membrane^40^, whereas some characterised Csls are localised to the Golgi apparatus^41^. The significance of the ER localisation of CSyGT is also presented in related work^36^. SOAP5 co-localised to the ER with SOAP1–4 and Förster resonance energy transfer (FRET) suggested protein–protein interactions with SOAP4. However, no interaction between SOAP5 and other SOAP enzymes was observed, Further experiments should investigate whether these interactions are conserved in other triterpenoid saponin-producing plants.

The binding of the first sugar moiety to the C-3 position of oleanolic acid in *Calendula officinalis* has been described as a decisive factor in its transport mechanism^42^. The researchers found that oleanolic acid glucosides are transported across the tonoplast by an active, energy-dependent carrier-mediated mechanism, whereas glucuronides are translocated primarily via facilitated diffusion^43^. Although no transcriptome data were available to confirm whether CSyGT homologues exist in *C. officinalis*, a report on the biosynthesis of the 3-*O-*glucuronide in the microsome^42^ suggests that CSyGT is involved in the 3-*O-*glucuronosylation of oleanolic acid in that species. These studies suggest that the 3-*O-*glucuronosylation of triterpenoids by CSyGT is involved in the intra- and intercellular translocation of triterpenoid saponins, and that ER localisation may not be the only decisive factor in the functional divergence of CSyGT from the CslM family. Nonetheless, the functional characterisation and subcellular localisation of CslM are needed to elucidate the evolutionary history of CSyGTs.

Finally, the discovery of the CSyGTs has elucidated the complete glycyrrhizin biosynthesis pathway and has enabled us to engineer a yeast strain that produces glycyrrhizin de novo from a simple sugar. Our strain expressed CYP72A63 from *Medicago truncatula* instead of the original glycyrrhizin biosynthetic enzyme CYP72A154 because CYP72A63 showed better catalytic activity in vivo in our previous study^44^. Jozwiak et al.^36^ also successfully synthesised glycyrrhizin by transient expression of the entire biosynthetic pathway in *N. benthamiana*, but they did not report the yield. Moreover, they reported that GuCsl (GuCSyGT) transferred a second glucuronic acid moiety with very low efficiency. No such catalytic activity was observed in our in vivo analysis with *S. cerevisiae*, and it is plausible that endogenous *N. benthamiana* UGT catalysed the second glucuronosylation. This work lays the foundation for the microbial production of glycyrrhizin and other high-value triterpenoid saponins. Further engineering, such as the pairing of CYPs and CPRs to increase catalytic activity, the manipulation of the mevalonate pathway to enhance the availability of the precursor 2,3-oxidosqualene and the introduction of a transporter that actively secretes the triterpenoid saponin product, may improve yields. We expect to be able to produce diverse, rare, natural and unnatural triterpenoid saponins using other biosynthetic enzymes from various plant sources and to promote the application of triterpenoid saponins in a variety of fields.

## Methods

### Chemicals

HPLC-grade methanol, 1-butanol, ascetic acid and water were purchased from Kishida Chemical, Osaka, Japan. Authentic standards of β-amyrin, (18β-)glycyrrhetinic acid, oleanolic acid, β-boswellic acid, kaempferol-3-*O-*monoglucuronide, genistein-7-*O-*glucuronide (Extrasynthese, Genay, France), soyasapogenol A, soyasapogenol B, liquiritigenin, liquiritin (Tokiwa Phytochemical, Chiba, Japan), soyasaponin I (Bb), soyasaponin III (Bb’) (ChromaDex, Irvine, CA, USA), ursolic acid, betulinic acid (Tokyo Chemical Industries, Tokyo, Japan), medicagenic acid (Apin Chemicals, Oxon, UK), kaempferol (Cayman Chemical, Ann Arbor, MI, USA), glycyrrhetinic acid-3-*O-*monoglucuronide (Nacalai Tesque, Kyoto, Japan), glycyrrhizin (Wako Pure Chemical Industries, Osaka, Japan), soyasapogenol B-3-*O-*monoglucuronide^13^, glycyrrhetinic acid-3-*O-*monoglucoside and glucoglycyrrhizin^45^ were dissolved in HPLC-grade methanol to 1 µM, and used for LC–MS analyses.

### Synthesis of first-strand cDNA

The roots of *Glycyrrhiza uralensis* strain 308–19 harvested in June 2011^24^ were used for RNA extraction. The total RNA was extracted using PureLink^®^ Plant RNA Reagent (Thermo Fisher Scientific, Waltham, MA, USA) from frozen plant tissues, treated with recombinant DNase I (RNase-free) (TaKaRa Bio, Shiga, Japan), and purified using the RNeasy^®^ Plant Mini Kit (QIAGEN, Hilden, Germany) following the RNA cleanup protocol^46^. First-strand cDNA was synthesised using the SMARTer™ RACE cDNA Amplification Kit (Clontech/TaKaRa Bio). The same strategy was used for synthesis of first-strand cDNA from *Lotus japonicus* (Gifu B-129). Total RNA was extracted from maturing seeds collected from greenhouse-cultivated *Glycine max* cv. Enrei using an RNeasy^®^ Plant Mini Kit (QIAGEN). Finally, first-strand cDNA was synthesised using the QuantiTect^®^ Reverse Transcription Kit (QIAGEN).

### Co-expression analysis of *CSyGTs*

For co-expression analyses of GmCSyGT1, “Option III. Find functional modules” was used for a network search in SoyNet (https://www.inetbio.org/soynet/)^23^. *Glyma.06G324300* (*GmCSyGT1*) was strongly co-expressed (*Z*-score threshold = 102.1) with genes involved in triterpenoid biosynthesis, including bAS (Glyma.07G001300), CYP72A61 (Glyma.08G238100), CYP93E1 (Glyma.08G350800), CYP72A69 (Glyma.15G243300), UGT73F2 (Glyma.07G254600), UGT73K5 (Glyma.16G033700), UGT73P10 (Glyma.01G046300), UGT73P2 (Glyma.11G05340), UGT91H4 (Glyma.08G181000) and UGT91H9 (Glyma.10G104700). The expression profile of each gene and GmCSyGT2–3 and GmCslM1–2 was retrieved from the Phytozome^47^. For GuCSyGT, a dataset of non-redundant UniGene sequences and their expression profiles was obtained from RNA-Seq of *G. uralensis* cDNA libraries (http://ngs-data-archive.psc.riken.jp/Gur/index.pl)^24^. In addition to UniGenes involved in triterpenoid saponin biosynthesis, UniGenes for *G. uralensis* CslM and GuUGT73F13 were identified using blast. Then, the corresponding expression levels were obtained from previous calculations in fragments per kilobase per million reads (FPKM) units. For LjCSyGT, the expression profiles of LjbAS (Lj3g3v2027430), LjCYP72A61 (Lj3g3v3776580), LjCYP93E1 (Lj1g3v3555800), and LjCSyGT (Lj3g3v1981230) were retrieved from Lotus Base (https://lotus.au.dk/)^25^.

After retrieving expression profiles from the three species, the expression values were normalised to *Z*-score values among the libraries, and hierarchical clustering was performed with Clustal 3.0^48^. The results were illustrated using Java TreeView 1.1.6r2^49^.

### Cloning of *CSyGTs* and *GmCslMs*

All primers, plasmids and yeast strains used or generated in this study are listed in Supplementary Tables 1, 2 and 3, respectively.

The full-length *GuCSyGT* was amplified by polymerase chain reaction (PCR) using PrimeSTAR Max DNA Polymerase (TaKaRa Bio) with primers 1 and 2 (Supplementary Table 1) from the first-strand cDNA of *G. uralensis*. The initial denaturation step (98 °C for 1 min) was followed by 35 cycles of 98 °C for 10 s, 55 °C for 5 s and 72 °C for 10 s. The amplified cDNA was cloned into pENTR™/D-TOPO^®^ (Thermo Fisher Scientific) to produce an entry clone. Similarly, the GuUGT73F13 entry clone was obtained using primers 19 and 20. The LjCSyGT entry clone was produced from first-strand cDNA of *L. japonicus* using primers 3 and 4.

Full-length *GmCSyGTs* (*GmCSyGT1, GmCSyGT2* and *GmCSyGT3*) and *GmCslMs* (*GmCslM1* and *GmCslM2*) were amplified by PCR using PrimeSTAR GXL DNA Polymerase (TaKaRa Bio) with primers 5 and 6, 7 and 8, 9 and 10, 11 and 12 and 13 and 14, respectively, from the first-strand cDNA. The amplified cDNAs were each cloned into pDONR™221 (Thermo Fisher Scientific) using Gateway™ BP Clonase™ II Enzyme Mix (Thermo Fisher Scientific) to produce the corresponding entry clones. The GuCSyGT and LjCSyGT cDNAs were also cloned into pDONR™221 using the primer pairs, 21 and 22, and 23 and 24, respectively, for transformation of *L. japonicus* hairy roots.

### Constructs for in vivo yeast assays

For galactose-inducible dual expression of *Arabidopsis thaliana* *UGD* and *CSyGTs*, the plasmid pESC-HIS(*GAL10/UGD;GAL1/GW*) (Supplementary Table 2, plasmid no. 3) was constructed as follows. The coding sequence of *UGD* was PCR-amplified from the cDNA clone RAFL09-33-I02 provided by RIKEN BRC through the National Bio-Resource Project of MEXT, Japan, using primers 15 and 16, and cloned into the *SpeI*-digested Gateway-compatible version of the pESC-HIS vector. CSyGT/CslM cDNAs were subsequently transferred into plasmid no. 3 using Gateway™ LR Clonase™ II Enzyme Mix (Thermo Fisher Scientific) to generate plasmid nos. 4–10, respectively. Plasmid no. 11 was generated by transferring the GuUGT73F13 cDNA from its entry vector. Plasmid nos. 12 and 13 were generated as above with an empty entry vector, for use as controls. The plasmid pESC-URA(*GAL10/CYP72A63;GAL1/GW*) for galactose-inducible dual expression of *Medicago truncatula CYP72A63*^44^ and UGTs was generated using the same cloning strategy with primers 17 and 18. Plasmid nos. 15 and 16 were generated through an LR clonase reaction of plasmid no. 14 and the entry vector of *G. uralensis UGT73P12* and *G. uralensis UGT73P12v*, respectively^11^. Plasmid nos. 17 and 18 were generated by transferring *G. uralensis* CYP88D6^50^ to the pELC^50^ plasmid and *Medicago truncatula* CYP72A63^44^ to the pYES-DEST52 (Invitrogen) plasmid, respectively.

### Generation of yeast strains

*Saccharomyces cerevisiae* INV*Sc*1 (*MATa his3Δ1 leu2 trp1–289 ura3–52/MAT his3Δ1 leu2 trp1–289 ura3–52*, Thermo Fisher Scientific) pre-transformed with the pYES3-ADH-OSC1^50^ plasmid for constitutive expression of *L. japonicus β-amyrin synthase* (*bAS*) driven by the *ADH1* promoter was transformed with plasmid no. 17 for galactose-inducible dual expression of *L. japonicus CPR1* and *CYP88D6* using the Frozen-EZ Yeast Transformation II Kit™ (Zymo Research, Irvine, CA, USA). The resulting yeast strain G0 was further transformed with plasmid no. 18 for galactose-inducible expression of *CYP72A63* to obtain the glycyrrhetinic acid-producing yeast strain GA. Strain GL was generated by transforming G0 with plasmid no. 15 and GLU with plasmid no. 16.

### In vivo yeast assay

The triterpenoid saponin aglycone-producing yeast strains, GA, SB^27^ and OA^28^ were transformed with plasmid nos. 4–10 to generate GA1–7, SB1–7 and OA1–7, respectively. In addition, GA was transformed with plasmid nos. 11 and 13 to generate GA8 and GA9. Plasmid nos. 13 and 4 were introduced to GLU to generate GLU1 and GLU2, respectively. Plasmid no. 12 was used to generate the negative controls, GA0, SB0, OA0 and GLU0. Transformants were selected on synthetic defined (SD) medium without tryptophan, leucine, uracil and histidine (-Trp, -Leu, -Ura and -His) for GAs and SBs, and SD (-Trp, -Leu and -His) medium for OAs. Each glycerol stock (50 µL) was inoculated into the corresponding 1-mL SD medium containing 2% glucose and pre-cultured at 30 °C for 24 h at 200 rpm. Yeast cells were pelleted by centrifugation, rinsed with fresh corresponding SD medium, resuspended in 5 mL of the corresponding 5-mL SD medium containing 2% galactose for induction and incubated at 30 °C for 5 days at 200 rpm. Yeast metabolites were extracted twice with 4-mL HPLC-grade 1-butanol, evaporated and resuspended in 400 µL of HPLC-grade methanol. The solution was filtered through a GL chromate disk 4 A filter (pore size: 0.2 µm; GL Sciences, Tokyo, Japan) and used for LC–MS analyses.

### Yeast-feeding assay

INV*Sc*1 was transformed with the plasmids 4–10 to generate FA1–7, respectively. Plasmid no. 12 was used to generate FA0 as a negative control. Transformants were selected on an SD (-His) plate. Each glycerol stock (300 µL) was inoculated into 6-mL SD medium containing 2% glucose and pre-cultured. After rinsing, yeast cells were resuspended in 6-mL SD medium containing 2% galactose and divided into equal 12 samples. To each 0.5-mL aliquot, 4.5-mL SD medium containing 2% galactose and 10 µM of each substrate dissolved in methanol (β-amyrin, glycyrrhetinic acid, soyasapogenol A, soyasapogenol B, oleanolic acid, medicagenic acid, ursolic acid, β-boswellic acid, betulinic acid, liquiritigenin, kaempferol and genistein) were added. Yeast cells were cultured and metabolites were extracted as described in the in vivo yeast assay.

### Combinatorial production of glycyrrhizin

Strain GL was transformed with plasmid nos. 4–6 to generate GL1–3, respectively. Plasmid no. 12 was used to generate GL0 as a negative control. Transformants were selected on SD (-Trp, -Leu, -URA and -His) plates. Yeast cells were cultured in the same manner as the in vivo yeast assay. Yeast cultures were pelleted by centrifugation, and metabolites were extracted twice with 4-mL HPLC-grade 1-butanol from the yeast pellet and culture media separately. The samples were subsequently prepared in the same way as the in vivo yeast assay. The amount of glycyrrhizin produced was quantified from the LC–MS peak areas from three independent biological replicates.

### Liquid chromatography–mass spectrometry

Extracted metabolites were detected using the ACQUITY UPLC/MS system (Waters, Milford, MA, USA) with an ACQUITY UPLC HSS C18 column (2.1 × 150-mm column and 2.1 × 5-mm VanGuard pre-column; particle size, 1.8 μm, Waters, Milford, MA, USA) and an ACQUITY TQ Detector (Waters) in electrospray ionisation negative-ion mode with selected-ion monitoring (SIM). The mobile phase was composed of 0.025% (v/v) acetic acid in water (solvent 1) and 0.025% (v/v) acetic acid in acetonitrile (solvent 2). Samples were separated via gradient elution with 30% solvent 2 for 6 min to 100% over 22 min (40% at 6 min, 50% at 18 min and 100% at 28 min) at a flow rate of 0.20 mL/min. The final condition was maintained for 3.5 min and returned to the initial condition, resulting in a total chromatography run time of 38.5 min. The sample manager and the column were kept at 15 °C and 30 °C, respectively. For MS detection, the capillary voltage was set to 2.5 kV, cone voltage to 80 V, extractor voltage to 3 V, source temperature to 150 °C, desolvation temperature to 350 °C, cone gas flow to 50 L/h and desolvation gas flow to 600 L/h. The quantities of extracted compounds of interest were determined from the peak areas using MassLynx software (Waters). The values used for SIM for each assay are listed in Supplementary Table 4.

### Analyses of *L. japonicus LjCSyGT* mutants

*Lotus japonicus CSyGT* mutants were obtained from the *LORE1*^31^ collection of Lotus Base^25^. Of 19 *LORE1* mutant lines, two (30006020 and 30115796) were selected based on the location of the *LORE1* insertion in the *CSyGT* sequence and the total number of *LORE1* insertions in other genes. After germination of seeds of the mutant lines, genomic DNA was extracted from their cotyledons. The insertion of *LORE1* in *CSyGT* was confirmed by PCR using GoTaq Colorless Master Mix (Promega, Madison, WI, USA) and primers 25 and 26 for 30006020, primers 27 and 28 for 30115796 and primer 29 for *LORE1*.

One month after germination, the roots of each mutant line were harvested, lyophilised and powdered using Multi-beads shocker (2000 rpm, 60 s, Yasui Kikai). Then, 5-mg samples were extracted three times with 1 mL of HPLC-grade methanol by 15 s of vortex-mixing and 20 min of sonication. Methanol was evaporated, and the precipitate was resuspended in 500 µL of methanol, filtered through a GL chromate disk 4A filter and used for LC–MS analyses.

### Functional complementation of *L. japonicus CSyGT* mutants

GuCSyGT, LjCSyGT and GmCSyGT1 cDNAs were transferred from the pDONR entry clones to the P35S:GFP-gw^51^ vector through an LR clonase reaction, generating plasmid nos. 19, 20 and 21, respectively. Hairy roots transformed with *GuCSyGT*, *LjCSyGT* and *GmCSyGT1* were generated using *Agrobacterium rhizogenes* LBA 1334 as described^52^. Briefly, seedlings of homozygous *LjCSyGT* mutant lines were placed in suspensions of *A. rhizogenes* harbouring plasmid nos. 19, 20 and 21, in a Petri dish, and cut at the base of the hypocotyl. The cut seedlings were placed in co-culture medium (1/2 B5 medium) and grown for 4 days at 21 °C, shielded from light with aluminium foil. Next, the plants were transferred to HRE medium and grown for 2 weeks (16 h of light at 25 °C/8 h of darkness at 23 °C). Hairy roots that emerged from the samples were tested for GFP fluorescence. Plants with transgenic hairy roots were transplanted to sterilised vermiculite pots supplied with B&D medium and grown for 1 month.

The transformed root lines were harvested and extracted as described previously. Two individual transformants with the same construct were combined to obtain sufficient sample. Three biologically independent replicates were performed.

### Subcellular localisation of LjCSyGT

*LjCSyGT stop-less* cDNA was cloned into pDONR™221 using the primer pairs, 23 and 24’. *LjCSyGT* cDNAs were transferred from pDONR entry clones to the pK7WGR2 or pK7RWG2^53^ vector through an LR clonase reaction, generating plasmids no. 22 and 23. Hairy roots transformed with *RFP-LjCSyGT* and *LjCSyGT-RFP* were generated using *Agrobacterium rhizogenes* AR1193, as described^52^. The plasmids ER-gk and G-gk were used as ER system and Golgi apparatus markers, respectively^54^. The localisation of RFP-LjCSyGT or LjCSyGT-RFP was examined with a confocal microscope (LSM710, Carl Zeiss) using an EC Plan-Neofluar objective lens. RFP fluorescence was excited at 543 nm and emission was detected at 548–680 nm. GFP fluorescence was excited at 488 nm and emission was detected at 493–538 nm. Microscopic images were taken with LSM710 (Carl Zeiss) and ZEN2011 SP3 (Carl Zeiss) and analysed with ZEN2.3 SP1 (Carl Zeiss).

Hairy roots of mutant lines transformed with either *RFP-LjCSyGT* or *LjCSyGT-RFP* were harvested and extracted as described previously for LC–MS analysis. Two individual transformants with the same construct were combined to obtain sufficient sample.

### Phylogenetic analyses

The predicted amino acid sequence data of *A. thaliana* and *G. max* were downloaded from Ensembl Plants (https://plants.ensembl.org/index.html)^55^, *Chenopodium quinoa* from ChenopodiumDB (https://www.cbrc.kaust.edu.sa/chenopodiumdb/)^56^, *G. uralensis* from *G. uralensis* GDB (http://ngs-data-archive.psc.riken.jp/Gur-genome/index.pl)^57^, *L. japonicus* from the Legume Information System (https://legumeinfo.org/)^58^ and *Panax ginseng* from the Ginseng Genome Database (http://ginsengdb.snu.ac.kr/)^59^. We searched for cellulose-synthase-like genes in six datasets based on a HMMER^25^ search using Pfam^60^ hidden Markov models (HMM) for Cellulose Synt PF03552. The other model, PF00535, was not used because only diverged subfamilies, CslA and CslC, contained the domain^61^. We ran hmmsearch against all six proteomes (*E*-value cut-off <1.0) and removed any duplicate sequences or isoforms. We also manually inspected protein hits and stripped those with low scores. Next, we ran hmmalign to assign residues to profile positions and removed sites with assignments with a <0.6 posterior probability. Finally, we removed sequences with a length <50% of the average sequence length and constructed a phylogenetic tree using RAxML version 8.2^62^. We ran RAxML auto model selection using the BIC criteria, and the model selected was JTT. We ran PROTGAMMAJTTX with 1000 rapid bootstrap analysis for the final tree model.

### Reporting summary

Further information on research design is available in the Nature Research Reporting Summary linked to this article.

## Supplementary information

Supplementary Figures and Tables

Peer Review File

Reporting Summary

## Data Availability

Nucleotide sequences of the genes reported in this work have been deposited in NCBI (Supplementary Table 5). Other data supporting the findings of this study are available from the corresponding author upon reasonable request. Source data are provided with this paper.

## Source data

Source data
