# Supplementary material for: A cellulose synthase-derived enzyme catalyses 3-O-glucuronosylation in saponin biosynthesis
Source: Nat Commun. 2020 Nov 16;11:5664. doi: 10.1038/s41467-020-19399-0 (PMC7669905; doi:10.1038/s41467-020-19399-0)
Supplement: Supplementary file 1 — Supplementary Figures and Tables [file 41467_2020_19399_MOESM1_ESM.pdf]

Supplementary Figures

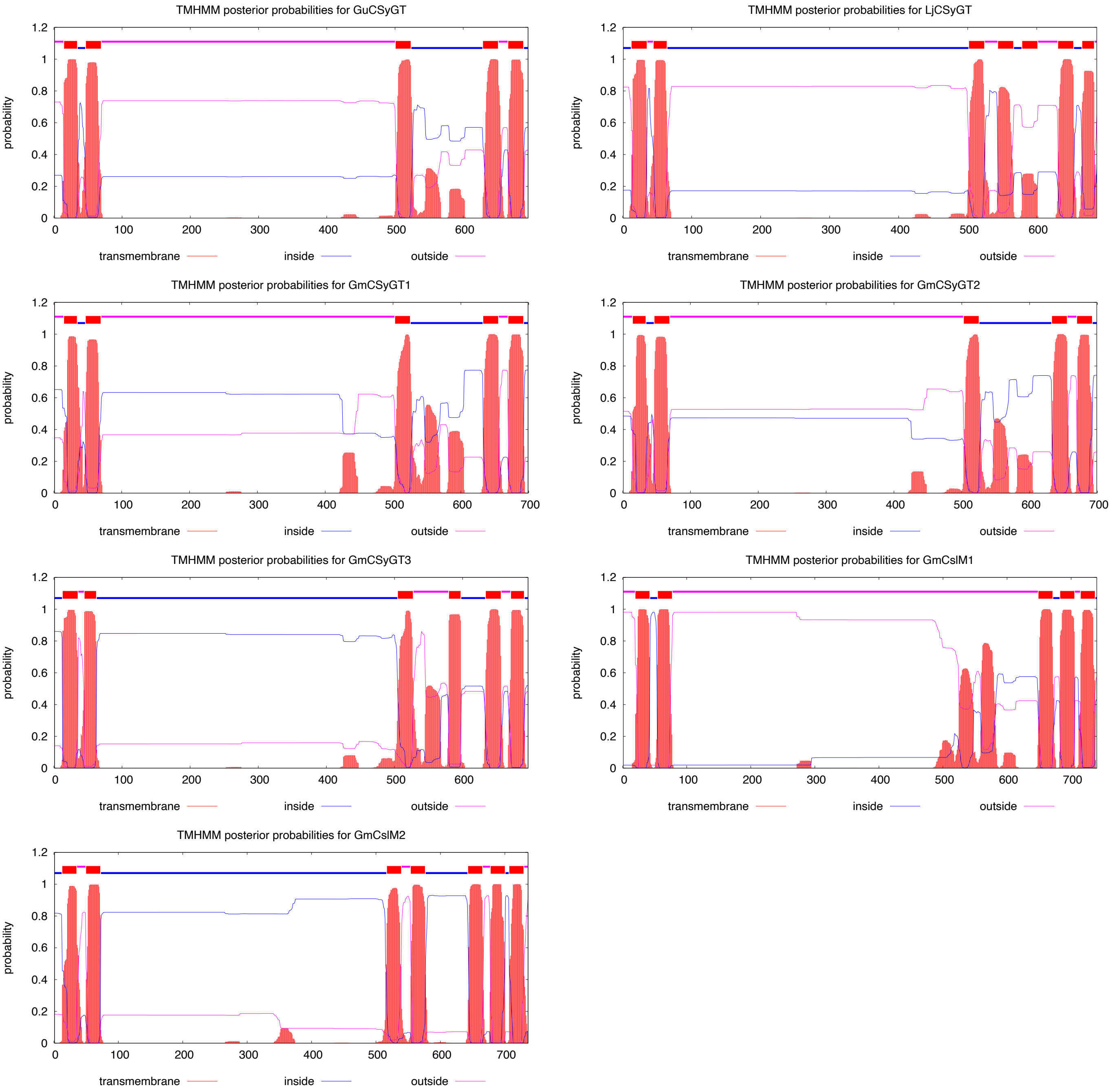

Supplementary Fig. 1: In silico analysis of the CSyGTs and GuCslM peptides. Transmembrane regions predicted by the program TMHMM.[26] The plot shows the posterior probabilities of inside, outside, and transmembrane helix for each amino acid residue. The N-best prediction obtained by calculating the total probability is shown at the top.

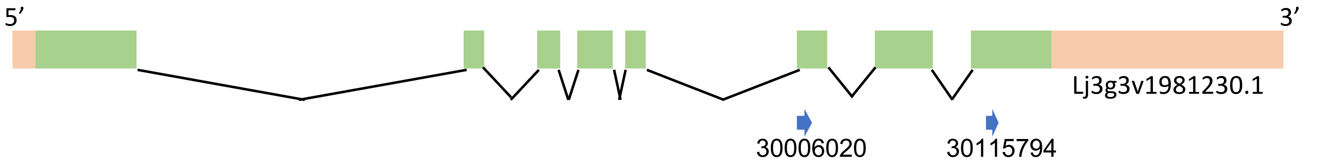

Supplementary Fig. 2: Schematic diagram of the LjCSyGT (Lj3g3v1981230) structure. The two mutant lines (30006020 and 30115796) were isolated form a Lj LORE1 insertion mutant population. Arrows indicate the positions of LORE1 insertions in the mutant lines.

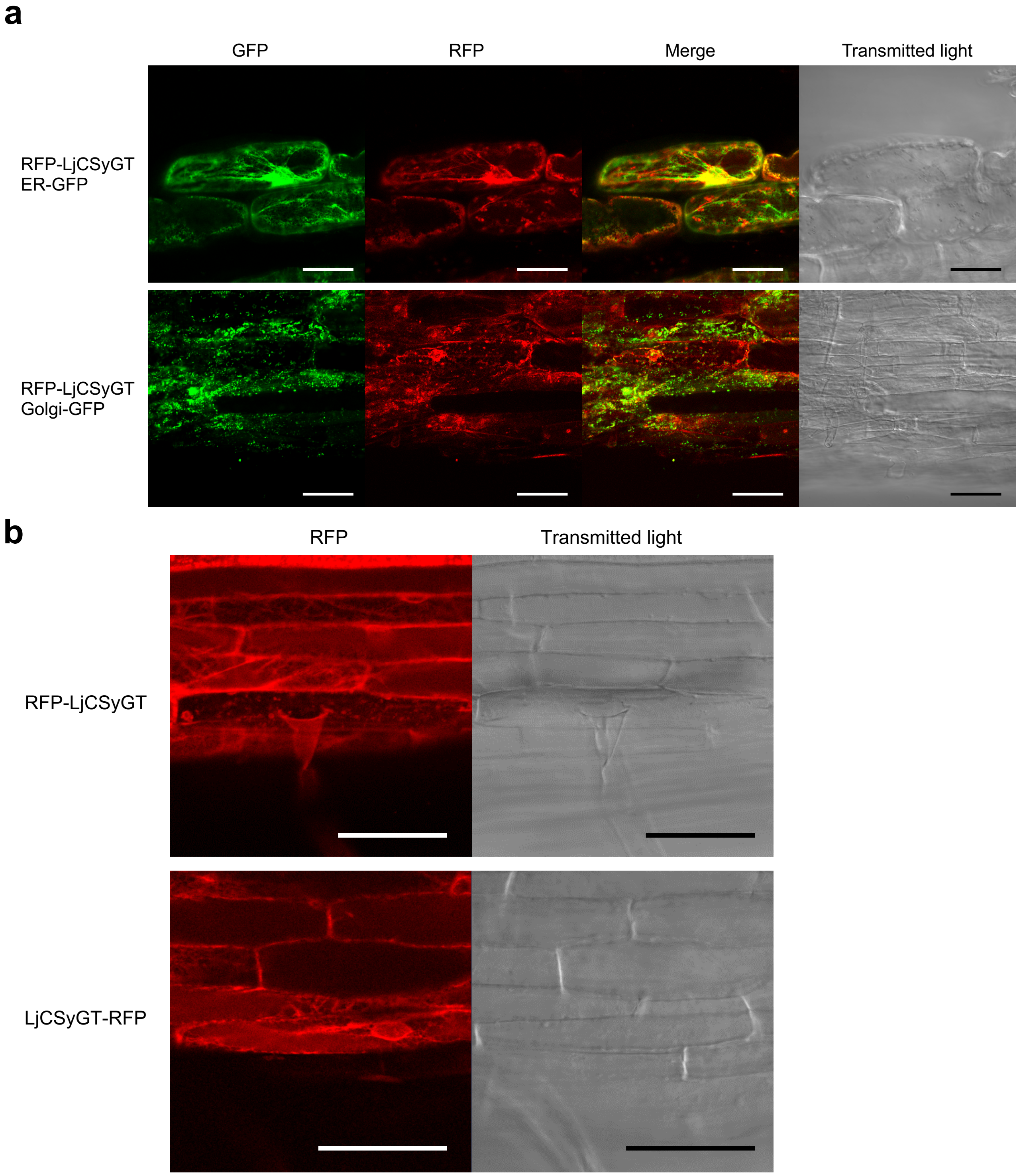

Supplementary Fig. 3: Subcellular localisation of LjCSyGT. a, Confocal image of RFP-LjCSyGT co-expressed in hairy roots of *L. japonicus* with either the ER or Golgi marker. Scale bars, 50 µm. **b**, Confocal image of RFP fused to either the N- or C-terminal of LjCSyGT (RFP-LjCSyGT or LjCSyGT-RFP) expressed in hairy roots of the *LjCSyGT* mutant of *L. japonicus.* Scale bars, 50 µm.

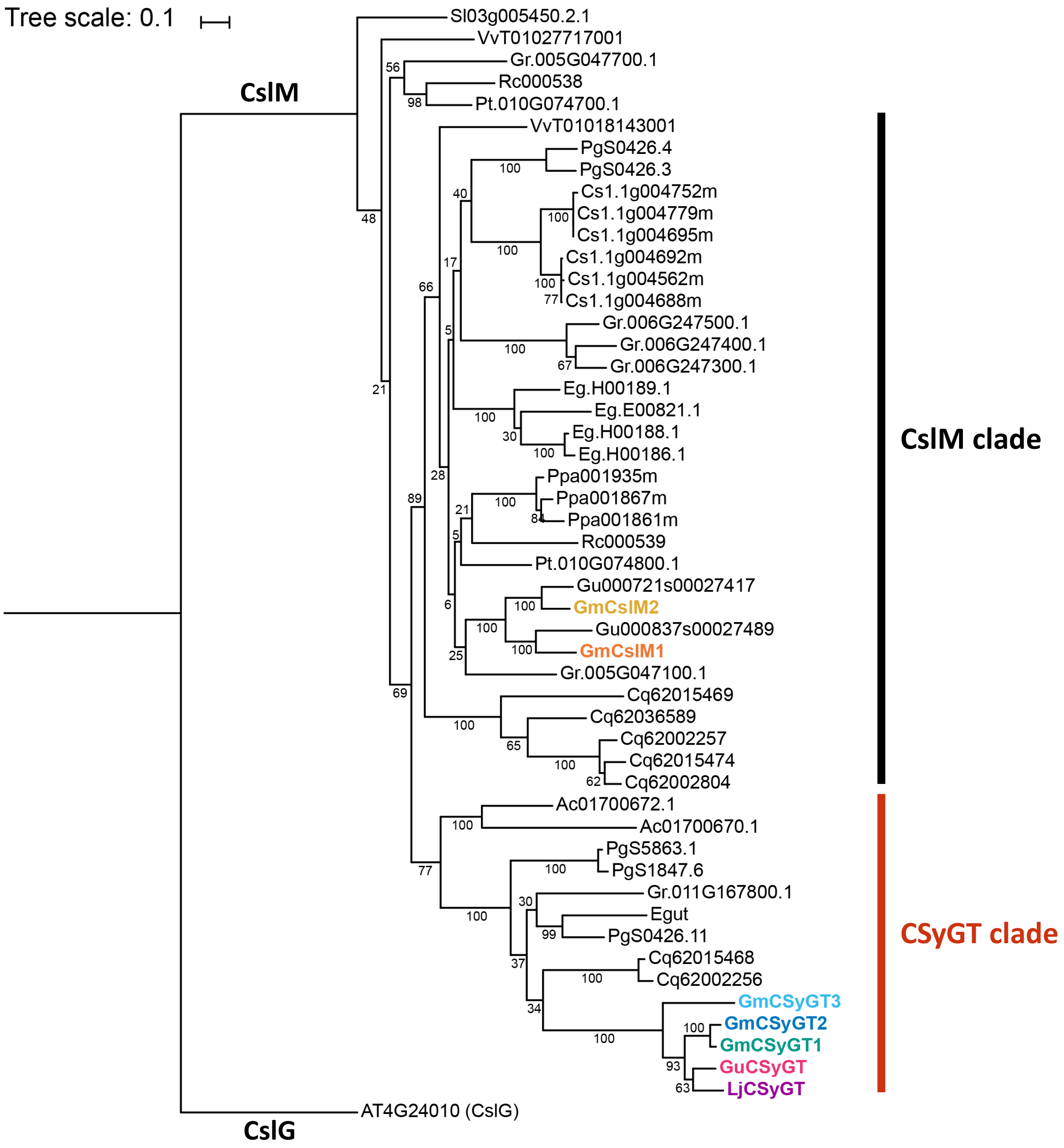

Supplementary Fig. 4: Phylogenetic analyses of the cellulose synthase M subfamily in angiosperms. Best-scoring maximum likelihood (ML) tree constructed using RAxML. Numbers indicate the bootstrap values (%) from 1,000 replicates. CSyGTs are clustered together, expanding from other CslMs. AT4G24010 (CslG) is included for rooting. Ac, *Aquilegia coerulea*; At, *Arabidopsis thaliana*; Cq, *Chenopodium quinoa*; Cs, *Citrus sinensis*; Eg, *Eucalyptus grandis*; Egut, *Erythranthe guttata*; Gm, *Glycine max*; Gr, *Gossypium raimondii*; Gu, *Glycyrrhiza uralensis*; Lj, *Lotus japonicus*; Pg, *Panax ginseng*; Pp, *Prunus persica*; Pt, *Populus trichocarpa*; Rc, *Ricinus communis*; Sl, *Solanum lycopersicum*; Vv, *Vitis vinifera*.

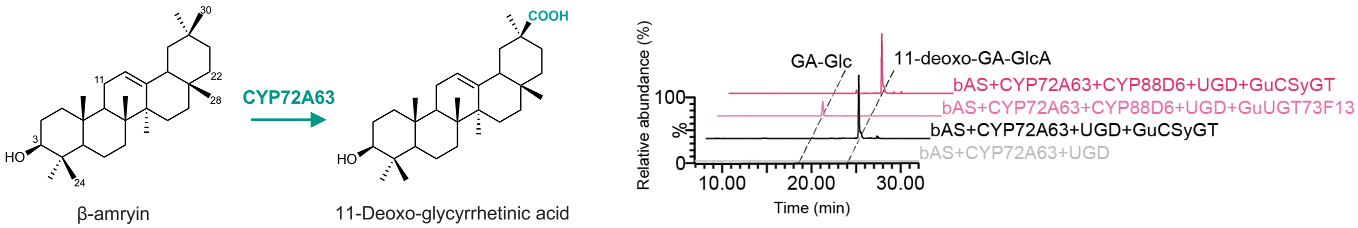

Supplementary Fig. 5: In vivo activity assay of GuCSyGTs using an 11-deoxo-glycyrrhetinic acid-producing yeast strain (bAS+CYP72A63). Catalytic activity of MtCYP72A63 against β-amyrin and an overlay of LC-MS chromatograms of products from transformed yeast strains with an *m/z* value of 631.4. GA, glycyrrhetinic acid; 11-deoxo-GA-GlcA, 11-deoxo-glycyrrhetinic acid monoglucuronide; GA-Glc, glycyrrhetinic acid-3-*O*-monoglucoside.

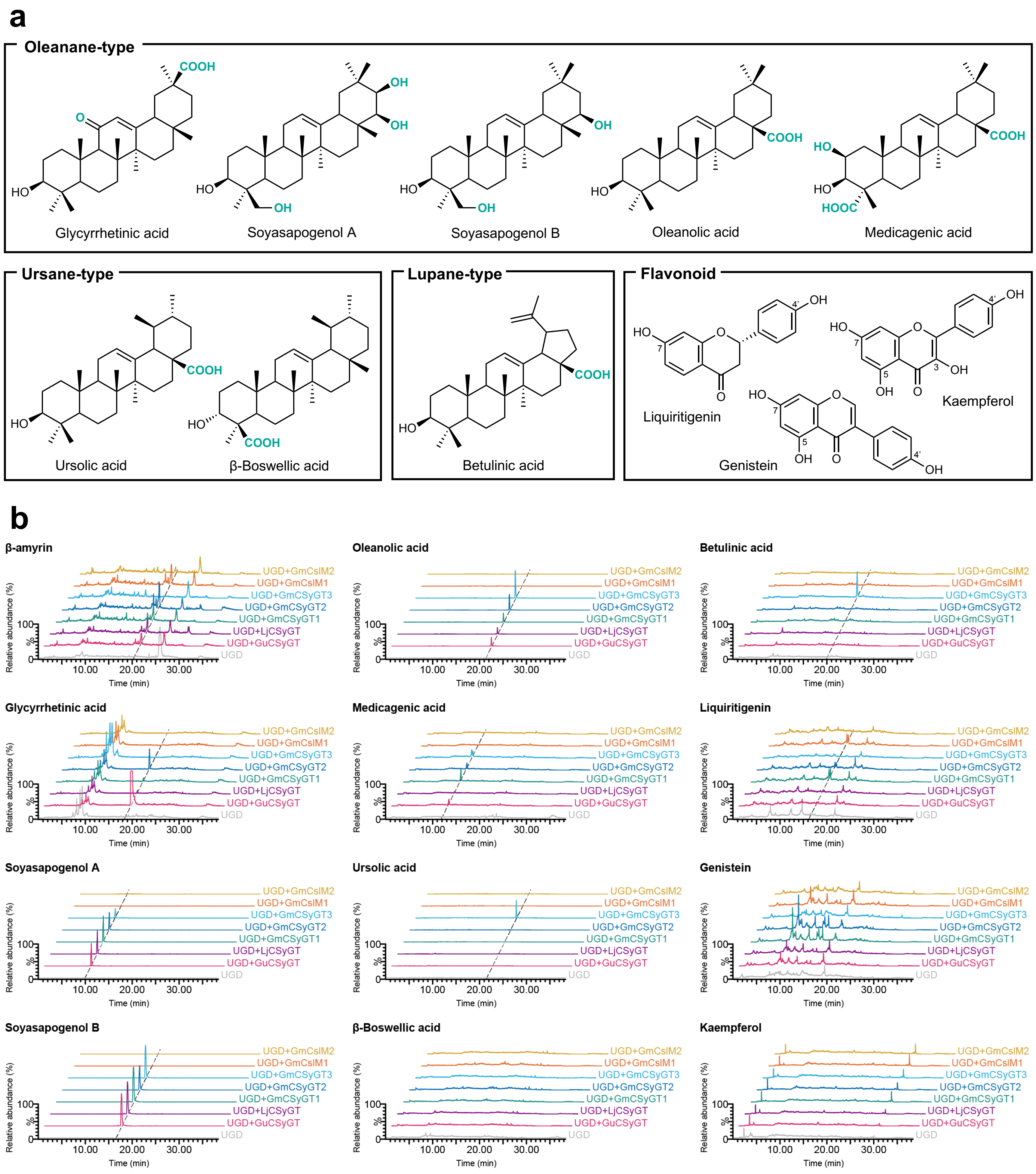

Supplementary Fig. 6: In vivo substrate-feeding assay of CSyGTs and GmCslMs. a, Chemical structures and classification of substrates. **b,** Overlays of LC-MS chromatograms of in vivo feeding assay extracts. Transformed strains (FA0–7) were fed with 10 µM substrate and cultured for 5 days after induction. Peaks of putative product monoglucuronides are indicated by dashed lines. All LC-MS chromatograms were selected based on the theoretical *m/z* values of the monoglucuronide products of interest (Supplementary Table 4).

Supplementary Tables

Supplementary Table 1: Primers used in this study

No.	Sequence (5´ to 3´)	Comment
1	CACCATGGCAAGCTTCACCCTTCACACAG	Cloning of *GuCSyGT*
2	TTATCCACTCTTGCTTTTCATGG	Cloning of *GuCSyGT*
3	CACCATGGCCAATTTCACTCTCCACAC	Cloning of *LjCSyGT*
4	CTACCCACTCTTCATTTTCATAG	Cloning of *LjCSyGT*
5	*aaaaagcaggct*ATGGCAATGTTCACGTACCACGTTG	Cloning of *GmCSyGT1*
6	agaaagctgggtCTATCCACTTTTGCTTTTCATTGTTACTATGGC	Cloning of *GmCSyGT1*
7	aaaaagcaggctATGGCAACGTTCACATACCACGTAG	Cloning of *GmCSyGT2*
8	agaaagctgggtCTATCCACTCTTGCTTTTCATTG	Cloning of *GmCSyGT2*
9	aaaaagcaggctATGGCGACCTTCCACACAGAAA	Cloning of *GmCSyGT3*
10	agaaagctgggtCTATTGCACCTTGCTTTTCATGG	Cloning of *GmCSyGT3*
11	aaaaagcaggctATGGAGGAGACTCTTTCTCTCAGTC	Cloning of *GmCslM1*
12	agaaagctgggtTTACACTTTCCTTAGTAGAGAATAG	Cloning of *GmCslM1*
13	aaaaagcaggctATGGAGACCCTTCCTCTGAATACC	Cloning of *GmCslM2*
14	agaaagctgggtCTAGTAGAGCATACTATGCATTCC	Cloning of *GmCslM2*
15	gggcggccgcactag**AAAA**ATGGTGAAGATATGTTGT	Cloning of *UGD*
16	atccatcgatactagTTAGGCAACGGCAGGCATGT	Cloning of *UGD*
17	cctcactaaagggcggccgc**AAAA**ATGGAAGTGTTTATGTTTC	Cloning of *CYP72A63*
18	catccttgtaatccatcgatTTACAGTTTATGCAAAATGATG	Cloning of *CYP72A63*
19	CACCATGGACGTTGCTGAAGAACAGCCACT	Cloning of GuUGT73F13
20	TTAGTTAAGCTGCTTAGAGTATCTCC	Cloning of GuUGT73F13
21	*aaaaagcaggct*ATGGCAAGCTTCACCCTTCAC	Cloning of *GuCSyGT*
22	*agaaagctgggt*TTATCCACTCTTGCTTTTCATGG	Cloning of *GuCSyGT*
23	*aaaaagcaggct*TTATGGCCAATTTCACTCTCCAC	Cloning of *LjCSyGT*
24	*agaaagctgggt*CCTACCCACTCTTCATTTTCATAG	Cloning of *LjCSyGT*
24'	*agaaagctgggt*CCCCACTCTTCATTTTCATAG	Cloning of *LjCSyGT stop-less*
25	GCGGCTAGGCTTGCGCTCAATTTT	Verifying *LORE1* insertion
26	TGCCTGTTTGGACACACAACCGTG	Verifying *LORE1* insertion
27	CGTTGGTTGCTGCTGGCCTAACAC	Verifying *LORE1* insertion
28	CCGCCAAAGAAGCAAACAATGTTCA	Verifying *LORE1* insertion
29	CCATGGCGGTTCCGTGAATCTTAGG	Verifying *LORE1* insertion

The sequences in lowercase represent vector sequence for In-Fusion® cloning (Takara Bio), the bold sequences were added to promote proper initiation of translation of downstream gene in yeast, the underlined sequences were added to facilitate unidirectional cloning of the product into pENTR™/D-TOPO® (Thermo Fisher Scientific), and the italicized lowercase sequences were added to allow BP clonase reaction (Thermo Fisher Scientific).

Supplementary Table 2: Plasmid vectors constructed for this study

No.	Construct	Backbone
1	pESC-HIS[*GAL1/GW*]	Seki et al., unpublished
2	pESC-URA[*GAL1/GW*]	Seki et al., unpublished
3	pESC-HIS[*GAL10/UGD;GAL1/GW*]	1
4	pESC-HIS[*GAL10/UGD;GAL1/GuCSyGT*]	3
5	pESC-HIS[*GAL10/UGD;GAL1/LjCSyGT*]	3
6	pESC-HIS[*GAL10/UGD;GAL1/GmCSyGT1*]	3
7	pESC-HIS[*GAL10/UGD;GAL1/GmCSyGT2*]	3
8	pESC-HIS[*GAL10/UGD;GAL1/GmCSyGT3*]	3
9	pESC-HIS[*GAL10/UGD;GAL1/GmCslM1*]	3
10	pESC-HIS[*GAL10/UGD;GAL1/GmCslM2*]	3
11	pESC-HIS[*GAL10/UGD;GAL1/GuUGT73F13*]	3
12	pESC-HIS[*GAL10/UGD*]	3
13	pESC-HIS[*GAL1/GuCSyGT*]	1
14	pESC-URA[*GAL10/CYP72A63;GAL1/GW*]	2
15	pESC-URA[*GAL10/CYP72A63;GAL1/UGT73P12*]	14
16	pESC-URA[*GAL10/CYP72A63;GAL1/UGT73P12v*]	14
17	pESC-LEU[*GAL10/CPR;GAL1/CYP88D6*]	pELC[10]
18	pYES-DEST52[GAL1/CYP72A63]	pYES-DEST52 (Invitrogen)
19	P35S:GFP-gw[*GuCSyGT*]	P35S:GFP-gw[16]
20	P35S:GFP-gw[*LjCSyGT*]	P35S:GFP-gw[16]
21	P35S:GFP-gw[*GmCSyGT1*]	P35S:GFP-gw[16]
22	pK7-RFP-LjCSyGT	pK7WGR2[18]
23	pK7-LjCSyGT-RFP	pK7RWG2[18]

Supplementary Table 3: Yeast strains generated in this study

Strain	Genotype	Source
INV*Sc*1	*MATa his3Δ1 leu2 trp1-289 ura3-52 MAT his3Δ1 leu2 trp1-289 ura3-52*	Thermo Fisher Scientific
G0	INV*Sc*1; pYES3[*ADH1/bAS*], pESC-LEU[*GAL10/CPR;GAL1/CYP88D6*]	10
GA	INV*Sc*1; pYES3[*ADH1/bAS*], pESC-LEU[*GAL10/CPR;GAL1/CYP88D6*], pYES-DEST52[*GAL1/CYP72A63*]	This study
GA0	GA; pESC-HIS[*GAL10/UGD*]	This study
GA1	GA; pESC-HIS[*GAL10/UGD;GAL1/GuCSyGT*]	This study
GA2	GA; pESC-HIS[*GAL10/UGD;GAL1/LjCSyGT*]	This study
GA3	GA; pESC-HIS[*GAL10/UGD;GAL1/GmCSyGT1*]	This study
GA4	GA; pESC-HIS[*GAL10/UGD;GAL1/GmCSyGT2*]	This study
GA5	GA; pESC-HIS[*GAL10/UGD;GAL1/GmCSyGT3*]	This study
GA6	GA; pESC-HIS[*GAL10/UGD;GAL1/GmCslM1*]	This study
GA7	GA; pESC-HIS[*GAL10/UGD;GAL1/GmCslM2*]	This study
GA8	GA; pESC-HIS[*GAL10/UGD;GAL1/GuUGT73F13*]	This study
GA9	GA; pESC-HIS[*GAL1/GuCSyGT*]	This study
SB	INV*Sc*1; pYES3[*ADH1/bAS*], pESC-LEU[*GAL10/CPR;GAL1/CYP93E3*], pYES-DEST52[*GAL1/CYP72A566*]	13
SB0	SB; pESC-HIS[*GAL10/UGD*]	This study
SB1	SB; pESC-HIS[*GAL10/UGD;GAL1/GuCSyGT*]	This study
SB2	SB; pESC-HIS[*GAL10/UGD;GAL1/LjCSyGT*]	This study
SB3	SB; pESC-HIS[*GAL10/UGD;GAL1/GmCSyGT1*]	This study
SB4	SB; pESC-HIS[*GAL10/UGD;GAL1/GmCSyGT2*]	This study
SB5	SB; pESC-HIS[*GAL10/UGD;GAL1/GmCSyGT3*]	This study
SB6	SB; pESC-HIS[*GAL10/UGD;GAL1/GmCslM1*]	This study
SB7	SB; pESC-HIS[*GAL10/UGD;GAL1/GmCslM2*]	This study
OA	INV*Sc*1; pYES3[*ADH1/bAS*], pYES-DEST52[*GAL1/CYP716A12*]	14
OA0	OA; pESC-HIS[*GAL10/UGD*]	This study
OA1	OA; pESC-HIS[*GAL10/UGD;GAL1/GuCSyGT*]	This study
OA2	OA; pESC-HIS[*GAL10/UGD;GAL1/LjCSyGT*]	This study
OA3	OA; pESC-HIS[*GAL10/UGD;GAL1/GmCSyGT1*]	This study
OA4	OA; pESC-HIS[*GAL10/UGD;GAL1/GmCSyGT2*]	This study
OA5	OA; pESC-HIS[*GAL10/UGD;GAL1/GmCSyGT3*]	This study
OA6	OA; pESC-HIS[*GAL10/UGD;GAL1/GmCslM1*]	This study
OA7	OA; pESC-HIS[*GAL10/UGD;GAL1/GmCslM2*]	This study
FA0	INV*Sc*1; pESC-HIS[*GAL10/UGD*]	This study
FA1	INV*Sc*1; pESC-HIS[*GAL10/UGD;GAL1/GuCSyGT*]	This study
FA2	INV*Sc*1; pESC-HIS[*GAL10/UGD;GAL1/LjCSyGT*]	This study
FA3	INV*Sc*1; pESC-HIS[*GAL10/UGD;GAL1/GmCSyGT1*]	This study
FA4	INV*Sc*1; pESC-HIS[*GAL10/UGD;GAL1/GmCSyGT2*]	This study
FA5	INV*Sc*1; pESC-HIS[*GAL10/UGD;GAL1/GmCSyGT3*]	This study
FA6	INV*Sc*1; pESC-HIS[*GAL10/UGD;GAL1/GmCslM1*]	This study
FA7	INV*Sc*1; pESC-HIS[*GAL10/UGD;GAL1/GmCslM2*]	This study
GL	G0; pESC-URA[*GAL10/CYP72A63;GAL1/UGT73P12*]	This study
GL0	GL; pESC-HIS[*GAL10/UGD*]	This study
GL1	GL; pESC-HIS[*GAL10/UGD;GAL1/GuCSyGT*]	This study
GL2	GL; pESC-HIS[*GAL10/UGD;GAL1/LjCSyGT*]	This study
GL3	GL; pESC-HIS[*GAL10/UGD;GAL1/GmCSyGT1*]	This study
GLU	G0; pESC-URA[*GAL10/CYP72A63;GAL1/UGT73P12v*]	This study
GLU0	GLU; pESC-HIS[*GAL10/UGD*]	This study
GLU1	GLU; pESC-HIS[*GAL1/GuCSyGT*]	This study
GLU2	GLU; pESC-HIS[*GAL10/UGD;GAL1/GuCSyGT*]	This study

Supplementary Table 4: Selected ion monitoring in electrospray ionization (ESI) negative ion mode for LC-MS analysis

**Strain**	**Molecular Formula**	***m/z***
β-amryin	$C_{30}H_{50}O$	425.4
β-amyrin monoglucuronide	$C_{36}H_{58}O_7$	601.4
glycyrrhetinic acid	$C_{30}H_{46}O_4$	469.3
glycyrrhetinic acid-3-*O*-monoglucoside	$C_{36}H_{56}O_9$	631.4
glycyrrhetinic acid-3-*O*-monoglucuronide	$C_{36}H_{54}O_{10}$	645.4
glucoglycyrrhizin	$C_{42}H_{64}O_{15}$	807.4
glycyrrhizin	$C_{42}H_{62}O_{16}$	821.4
soyasapogenol A	$C_{30}H_{50}O_4$	473.4
soyasapogenol A monoglucuronide	$C_{36}H_{58}O_{10}$	649.4
soyasapogenol B	$C_{30}H_{50}O_3$	457.4
soyasapogenol B-3-*O*-monoglucuronide	$C_{36}H_{58}O_9$	633.4
soyasaponin Bb	$C_{48}H_{78}O_{18}$	941.5
soyasaponin Bb’	$C_{42}H_{68}O_{14}$	795.5
oleanolic acid	$C_{30}H_{48}O_3$	455.4
oleanolic acid monoglucuronide	$C_{36}H_{56}O_9$	631.4
medicagenic acid	$C_{30}H_{46}O_6$	501.3
medicagenic acid monoglucuronide	$C_{36}H_{54}O_{12}$	677.4
ursolic acid	$C_{30}H_{48}O_3$	455.4
ursolic acid monoglucuronide	$C_{36}H_{56}O_9$	631.4
β-boswellic acid	$C_{30}H_{48}O_3$	455.4
β-boswellic acid monoglucuronide	$C_{36}H_{56}O_9$	631.4
betulinic acid	$C_{30}H_{48}O_3$	455.4
betulinic acid monoglucuronide	$C_{36}H_{56}O_9$	631.4
liquiritigenin	$C_{15}H_{12}O_4$	255.1
liquiritigenin monoglucuronide	$C_{21}H_{20}O_{10}$	431.1
kaempferol	$C_{15}H_{10}O_6$	285.0
kaempferol-3-*O*-monoglucuronide	$C_{21}H_{18}O_{12}$	461.1
genistein	$C_{15}H_{10}O_5$	269.1
genistein-7-*O*-monoglucuronide	$C_{21}H_{18}O_{11}$	445.1

Supplementary Table 5. Accession numbers of enzymes used in this study

Enzyme	Organism of origin	GenBank Accession No.
GuCSyGT	*Glycyrrhiza uralensis*	LC500232
LjCSyGT	*Lotus japonicus*	LC500233
GmCSyGT1	*Glycine max*	LC500227
GmCSyGT2	*Glycine max*	LC500228
GmCSyGT3	*Glycine max*	LC500229
GmCslM1	*Glycine max*	LC500230
GmCslM2	*Glycine max*	LC500231
GuUGT73F13	*Glycyrrhiza uralensis*	LC314774
