# Supplementary material for: A cellulose synthase-derived enzyme catalyses 3-O-glucuronosylation in saponin biosynthesis
Source: Nat Commun. 2020 Nov 16;11:5664. doi: 10.1038/s41467-020-19399-0 (PMC7669905; doi:10.1038/s41467-020-19399-0)
Supplement: Supplementary file 3 — Reporting Summary [file 41467_2020_19399_MOESM3_ESM.pdf]

nature research

Corresponding author(s): Toshiya Muranaka, Masao Ishimoto

Last updated by author(s): Oct 2, 2020

Reporting Summary

Nature Research wishes to improve the reproducibility of the work that we publish. This form provides structure for consistency and transparency in reporting. For further information on Nature Research policies, see our Editorial Policies and the Editorial Policy Checklist.

Statistics

For all statistical analyses, confirm that the following items are present in the figure legend, table legend, main text, or Methods section.

n/a	Confirmed	
☐	☒	The exact sample size (*n*) for each experimental group/condition, given as a discrete number and unit of measurement
☐	☒	A statement on whether measurements were taken from distinct samples or whether the same sample was measured repeatedly
☒	☐	The statistical test(s) used AND whether they are one- or two-sided<br>*Only common tests should be described solely by name; describe more complex techniques in the Methods section.*
☒	☐	A description of all covariates tested
☒	☐	A description of any assumptions or corrections, such as tests of normality and adjustment for multiple comparisons
☐	☒	A full description of the statistical parameters including central tendency (e.g. means) or other basic estimates (e.g. regression coefficient) AND variation (e.g. standard deviation) or associated estimates of uncertainty (e.g. confidence intervals)
☒	☐	For null hypothesis testing, the test statistic (e.g. *F*, *t*, *r*) with confidence intervals, effect sizes, degrees of freedom and *P* value noted<br>*Give P values as exact values whenever suitable.*
☒	☐	For Bayesian analysis, information on the choice of priors and Markov chain Monte Carlo settings
☒	☐	For hierarchical and complex designs, identification of the appropriate level for tests and full reporting of outcomes
☒	☐	Estimates of effect sizes (e.g. Cohen's *d*, Pearson's *r*), indicating how they were calculated

Our web collection on statistics for biologists contains articles on many of the points above.

Software and code

Policy information about availability of computer code

Data collection	UPLC/MS: Acquity UPLC/MS system (Waters), Acquity TQ Detector (Waters), MassLynx (Waters)
Data analysis	Multiple sequence alignment: HMMER (3.1b2)<br>Phylogenetic analysis: RAxML (8.2.12)<br>Prediction of transmembrane regions: TMHMM Server (v. 2.0)<br>Gene coexpression analyses: Clustal 3.0, Java TreeView (1.1.6r2)<br>Quantification data analysis to make figures: Microsoft Excel (Mac 16.16.18)<br>Editing of figures: Adobe Illustrator 2019, iTOL (v5), ChemDraw Professional 16.0

For manuscripts utilizing custom algorithms or software that are central to the research but not yet described in published literature, software must be made available to editors and reviewers. We strongly encourage code deposition in a community repository (e.g. GitHub). See the Nature Research guidelines for submitting code & software for further information.

Data

Policy information about availability of data

All manuscripts must include a data availability statement. This statement should provide the following information, where applicable:

- Accession codes, unique identifiers, or web links for publicly available datasets
- A list of figures that have associated raw data
- A description of any restrictions on data availability

The nucleotide sequences of genes reported in this work have been deposited in NCBI (accessions LC500232, LC500233, LC500227, LC500228, LC500229, LC500230, LC500231, LC314774LC425153 and LC425154).

Field-specific reporting

Please select the one below that is the best fit for your research. If you are not sure, read the appropriate sections before making your selection.

- [x] Life sciences
- [ ] Behavioural & social sciences
- [ ] Ecological, evolutionary & environmental sciences

For a reference copy of the document with all sections, see nature.com/documents/nr-reporting-summary-flat.pdf

Life sciences study design

All studies must disclose on these points even when the disclosure is negative.

Sample size	No sample size calculation was performed in advance. Samples sizes are determined according to comparable experiments in previously articles.
Data exclusions	No data were excluded in the experiment.
Replication	Quantification data sets were performed with three independent biological replicates.
Randomization	No experiments were carried out in randomized conditions because randomization was not seriously relevant in this study.
Blinding	No investigations were blinded because blinding was not seriously relevant to our non-clinical experiments.

Reporting for specific materials, systems and methods

We require information from authors about some types of materials, experimental systems and methods used in many studies. Here, indicate whether each material, system or method listed is relevant to your study. If you are not sure if a list item applies to your research, read the appropriate section before selecting a response.

Materials & experimental systems

n/a	Involved in the study	
x		Antibodies
	x	Eukaryotic cell lines
x		Palaeontology and archaeology
x		Animals and other organisms
x		Human research participants
x		Clinical data
x		Dual use research of concern

Methods

n/a	Involved in the study	
x		ChIP-seq
x		Flow cytometry
x		MRI-based neuroimaging

Eukaryotic cell lines

Policy information about cell lines

Cell line source(s)	Lotus japonicus mutant lines (30006020 and 30115796) were obtained from the LORE1 insertion mutant resource (Małolepszy et al. 2016). Saccharomyces cerevisiae lines were generated using the commercially available INVSc1 strain (Thermo Fisher Scientific) as a host.
Authentication	The insertion of LORE1 in the target site was confirmed by PCR of genomic DNA extracted from cotyledons of each line. Yeast strains were authenticated by auxotrophic phenotyping.
Mycoplasma contamination	Not tested.
Commonly misidentified lines (See ICLAC register)	n/a
