# Supplementary material for: A cellulose synthase-derived enzyme catalyses 3-O-glucuronosylation in saponin biosynthesis
Source: Nat Commun. 2020 Nov 16;11:5664. doi: 10.1038/s41467-020-19399-0 (PMC7669905; doi:10.1038/s41467-020-19399-0)
Supplement: Supplementary file 2 — Peer Review File [file 41467_2020_19399_MOESM2_ESM.pdf]

Editorial Note: This manuscript has been previously reviewed at another journal that is not operating a transparent peer review scheme. The manuscript was considered suitable for publication without further review at *Nature Communications.*
